# *Coptis rhizome* extract influence on *Streptococcus pneumoniae* through autolysin activation

**DOI:** 10.1186/s13568-024-01736-x

**Published:** 2024-07-04

**Authors:** Eon-Bee Lee, Kyubae Lee

**Affiliations:** 1 Laboratory of Veterinary Pharmacokinetics and Pharmacodynamics, College of Veterinary Medicine, Kyungpook National University, Daegu, 41566 Republic of Korea; 2 Veterinary Drugs and Biologics Division, Animal and Plant Quarantine Agency, Gimcheon, 39660 Republic of Korea; 3 Department of Biomedical Materials, Konyang University, Daejeon, 35365 Republic of Korea

**Keywords:** *Coptis rhizome*, *Streptoccus pneumonia*, Autolysis, lytA, Antimicrobial activity, Biofilm formation

## Abstract

This study investigated the antibacterial properties of *Coptis rhizome*, a plant traditionally used for respiratory infections, against *Streptoccus pneumonia* (*S. pneumoniae*), for which there has been minimal empirical evidence of effectiveness. The study particularly examined autolysis, indirectly associated with antibacterial resistance, when using *Coptis rhizome* for bacterial infections. In our methodology, *Coptis rhizome* was processed with ethanol and distilled water to produce four different extracts: CRET30, CRET50, CRET70, and CRDW. The antibacterial activity of these extracts were tested through Minimum Inhibitory Concentration (MIC) assays, disk diffusion tests, and time-kill assays, targeting both standard (ATCC 49619) and resistant (ATCC 70067) strains. The study also evaluated the extracts’ biofilm inhibition properties and monitored the expression of the lyt gene, integral to autolysis. The results prominently showed that the CRET70 extract demonstrated remarkable antibacterial strength. It achieved an MIC of 0.125 µg/mL against both tested *S. pneumoniae* strains. The disk diffusion assay recorded inhibition zones of 22.17 mm for ATCC 49619 and 17.20 mm for ATCC 70067. Impressively, CRET70 resulted in a 2-log decrease in bacterial numbers for both strains, showcasing its potent bactericidal capacity. The extract was also effective in inhibiting 77.40% of biofilm formation. Additionally, the significant overexpression of the lytA gene in the presence of CRET70 pointed to a potential mechanism of action for its antibacterial effects. The outcomes provided new perspectives on the use of *Coptis rhizome* in combating *S. pneumoniae*, especially significant in an era of escalating antibiotic resistance.

## Introduction

Medicinal plants have been identified and utilized in traditional medical practices for their potential health advantages and healing qualities (Gurib-Fakim 2006). They demonstrate a range of activities and medicinal properties because of the variety of bioactive compounds present in them (Ramawat and Mérillon 2008). These activities can range from anti-inflammatory and antioxidant to antimicrobial and analgesic (Rios and Recio 2005). *Coptis rhizome*, also known as Huang Lian in traditional Chinese medicine, is the dried rhizome (root) of the *Coptis chinensis* plant (Lee et al. 1991). Several therapeutic benefits of berberine, the active compound in *Coptis chinensis*, have also been recorded. These include treating experimental colitis (Zhou and Mineshita 2000), exhibiting anti-inflammatory effects (Kuo et al. 2004) and aiding in the control of diabetes (Zhou et al. 2007).

*Streptococcus pneumonia*, often referred to as pneumococcus, is a bacterium that can cause various infections in humans, including pneumonia, otitis media, sinusitis, and invasive diseases like bacteremia and meningitis (Feldman and Anderson 2014; Weiser et al. 2018). It is a significant pathogen responsible for a range of respiratory and systemic illnesses, especially in vulnerable populations such as young children, the elderly, and individuals with weakened immune systems (Hoffman et al. 2003).

Cell wall biosynthesis is a fundamental process in bacterial growth and maintenance, involving the intricate construction and regulation of the cell wall (Zhong and Ye 2007). Bacterial cell walls consist primarily of peptidoglycan, a complex polymer made of sugars and amino acids (Silhavy et al. 2010). The autolysin protein, encoded by the lytA gene, plays a pivotal role in bacterial cell wall dynamics, particularly in species like *S. pneumonia* (Li et al. 2015). This enzyme is crucial for the controlled degradation and remodeling of the peptidoglycan, a fundamental component of the bacterial cell wall. By cleaving peptidoglycan bonds, the autolysin allows for the insertion of new peptidoglycan units during cell growth and division, maintaining cell shape and integrity (Standish et al. 2014). Moreover, autolysins can release cell wall fragments or muropeptides, which can serve as signaling molecules and impact host immune responses (Afshar et al. 2020). In pathogenic bacteria like *S. pneumoniae*, autolysin proteins also contribute to virulence, colonization, tissue invasion, and evasion of host defenses (Tomasz et al. 1988). Furthermore, autolysin activity can influence antibiotic susceptibility, potentially making them valuable targets for antibacterial therapies.

When autolysin proteins, such as lytA, are overexpressed, a disruption in the equilibrium of cell wall biosynthesis occurs, leading to potential bacterial death (López et al. 2004). Excessive autolysin activity results in the hyperactive cleavage of peptidoglycan bonds, causing extensive damage to the cell wall structure. The weakened cell wall loses its ability to maintain cell shape and withstand internal turgor pressure, ultimately leading to cell lysis or bursting. As a result, the internal contents of the bacterium are released into the surrounding environment, resulting in bacterial death.

Numerous studies have highlighted the effectiveness of *Coptis rhizome* in combating various microbial pathogens, showcasing its strong antimicrobial capabilities. Historically, it has been used for treating respiratory infections (Aires et al. 2023). Yet, there has been a lack of research investigating its effects against *S. pneumoniae*, particularly strains resistant to conventional treatments. To date, no studies have specifically concentrated on how the extract impacts the activity of autolysins in *S. pneumonia*, which could open up new avenues for therapeutic approaches. The aim of this study is to explore the potential of *Coptis rhizome* extract in influencing autolysin activation, offering a fresh perspective in the development of antibacterial treatments, especially for *S. pneumonia* strains that are resistant to current antibiotics.

## Materials and methods

### Bacterial strains and culture conditions

The strains ATCC 49619 and ATCC 70067 of *S. pneumoniae* were acquired from the American Type Culture Collection (ATCC) (Manassas, Virginia, USA). These strains were grown in Brain Heart Infusion (BHI) medium, which was sourced from the BD company (New Jersey, USA). The cultures were incubated overnight at 37 °C in a 5% CO_2_, using BHI agar. Notably, the ATCC 70067 strain exhibits resistance to antibiotics such as penicillin, tetracycline, and erythromycin, but remains sensitive to rifampin. To ensure consistent growth, the *S. pneumoniae* was subcultured multiple times in BHI medium.

### Plant extraction procedure

*Coptis rhizome* samples were obtained from herbal markets (Andong, Korea). These plants were carefully cleaned with tap water to remove dust and other extraneous substances they may have gathered from their natural surroundings. After ensuring they were free of dust, the roots were left to dry in a shaded area of the botany lab for 20 days. The dried roots were then ground into a fine powder using an electric blender. This powder was further refined by passing it through a kitchen strainer to be used for extraction processes.

For the extraction, 20 g of this powdered plant material was placed in a 200 mL conical flask to which 100 mL of various solvents (30%, 50%, 70% ethanol, or distilled water) were added individually. The flask was then sealed with aluminum foil and placed on a shaker for 24 h. This process, conducted at 150 revolutions per minute, ensured thorough mixing and efficient extraction of active compounds into the solvents. The extract was then filtered using muslin cloth, Whatman no 1 filter paper, and finally a vacuum and pressure pump (AP-9925 Auto Science). The solvent was removed from the extract using a rotary vacuum evaporator RE52 at a water bath temperature of 50 °C, and the remaining residues were collected for experimental use. The solvent-free plant extracts were abbreviated as shown in Table 1 and were prepared at a concentration of 100 mg/mL by dissolving them in their respective solvents, either ethanol or distilled water.

**Table 1:** Extracted *Coptis rhizome* using various solvents

No.	Plant	Solvent	Abbreviation
1	*Coptis rhizome*	Ethanol 30%	CRET30
2	*Coptis rhizome*	Ethanol 50%	CRET50
3	*Coptis rhizome*	Ethanol 70%	CRET70
4	*Coptis rhizome*	Distilled water	CRDW

### Disk diffusion

To assess the antibacterial properties of the extracts, a disk diffusion method was employed, following previously published protocols (Biemer 1973), with a slight modification in the concentration of extracts (6 mg per disc). 0.5 McFarland bacterial suspension of *S. pneumoniae* was prepared in BHI. This suspension was then evenly spread across BHI agar using a sterile cotton swab. Discs were placed onto the agar media. Rifampin at 5 µg was used as a positive control since *S. pneumoniae* strains are susceptible to Rifampin, producing an inhibition zone typically measuring 25–30 mm (CLSI 2017). The media were subsequently incubated for 20 h at 37 °C with 5% CO_2_. The diameter of the inhibition zones observed the next day was measured by using digimatic caliper (Mitutoyo, kanakawa, Japan) to assess antimicrobial activity. Extracts with inhibition zones greater than 20 mm were selected for further analysis in time-kill assays and bacterial lysis experiments.

### MIC determination

The Minimum Inhibitory Concentration (MIC) of plant extracts including CRET30, CRET50, CRET70, and CRDW against *S. pneumoniae* ATCC 49619 and ATCC 70067 was determined using a two-fold serial dilution method, with concentrations ranging from 0.03125 to 64 µg/mL, following the Clinical and Laboratory Standards Institute (CLSI) guidelines (CLSI 2017). After inoculation, the plates were incubated at 37 °C for 24 h. The MIC was identified as the lowest concentration of the plant extract that visibly prevented bacterial growth in the medium. To confirm the results, a microplate reader (Versamax™, Idaho Emmett, ID, USA) was utilized.

### Time kill assay

In vitro time-kill experiments were conducted to evaluate the efficacy of CRET70 against *S. pneumoniae* ATCC 49619 and ATCC 70067, following the guidelines outlined by CLSI (CLSI 2017). Initially, the bacterial concentration was adjusted to a final inoculum of 1.6 × 10^6^ cfu/mL. Subsequently, these bacterial cultures were exposed to varying concentrations of CRET70, ranging from 1× to 4× MIC. As a positive control, Rifampin was added at a final concentration of 5 µg/mL, while a negative control consisted of bacterial suspension without any additional treatment. To establish control growth curves, bacterial cultures were also cultivated in BHI without any interventions. Bacterial counts were conducted at various time points, including 0, 1, 2, 4, 8, 12, and 24 h of incubation. Following this, the cultures were further incubated for an additional 24 h at 37 °C on BHI agar plates.

### Bacterial lysis

For all bacterial lysis tests conducted in this study, ATCC 49619 and ATCC 70067 were the selected bacterial strains. Furthermore, we used CRET70 at various concentrations, which showed the highest effectiveness among the samples. Bacterial lysis was assessed by measuring absorbance at 595 nm using an Epoch microplate reader (Winooski, VT, USA). To explain the procedure briefly, a bacterial suspension with a density of 0.5 McFarland was prepared in BHI. Subsequently, 90 µL of this suspension was dispensed into each well of a 96-well flat bottom plate. We then mixed 10 µL of the plant extract with the 90 µL bacterial suspension to achieve final concentrations of 1MIC, 2MIC, and 4MIC. A negative control group was included, consisting of bacterial suspension without any additions. All the experimental groups were conducted in triplicates. The plate was sealed with an optical adhesive seal and placed in the Epoch microplate reader, which was set to a kinetic loop mode with moderate shaking at 60 rpm for 5 min, followed by 10 min without shaking. The plate was incubated for 20 h, and absorbance readings at 595 nm were recorded every hour.

### Biofilm inhibition activity

The antibiofilm evaluation was carried out following a previously published method, with minor adjustments (Famuyide et al. 2019). To summarize, 50 µL of *S. pneumoniae* adjusted to a 0.5 McFarland standard was introduced into 5 mL of BHI. This enriched suspension was subsequently incubated at 37 °C in a 5% CO_2_ environment for 5 h. Following this, the bacterial suspension was standardized to a 0.5 McFarland standard by adding BHI. Afterward, 1 mL of the standardized suspension was transferred into 1.5 mL centrifuge tubes and centrifuged at 3000 rpm for 3 min. The supernatant was removed, and the bacterial cells underwent two washes with PBS 1× before being resuspended in 1 mL of BHI and homogenized.

Subsequently, 5 µL of this suspension was mixed with 895 µL of lysed-sheep blood. A 100 µL volume of plant extract (including CRET30, CRET50, CRET70, and CRDW) was combined with the lysed-sheep blood that had been inoculated and vortexed for thorough mixing. Following this, 50 µL of these mixtures were transferred into a 96-well flat-bottom plate and incubated at 37 °C in a 5% CO_2_ environment for 20 h. On the subsequent day, the plate was washed twice with PBS 1×, gently tapped on a paper towel, and air-dried for 15 min. Subsequently, 50 µL of a 0.1% crystal violet solution was added to each well and allowed to incubate for 15 min. The plate was then rinsed twice with PBS 1× and air-dried for another 15 min. The crystal violet that had adhered to the bacterial cell wall was dissolved in 120 µL of cold absolute ethanol with gentle mixing. Finally, 100 µL of the dissolved crystal violet was transferred to a fresh 96-well flat-bottom plate for absorbance measurement at 594 nm. All experiments were conducted in triplicate. A blank control was created using lysed-sheep blood, while a positive control involved mixing bacterial suspension in lysed-sheep blood with mouthwash. The negative control consisted of bacterial suspension in lysed-sheep blood.

### RNA extractions and relative gene expression

*Streptoccus pneumonia* cells obtained from ATCC 49619 and ATCC 70067 were subjected to treatment with CRET70 at different concentrations (1MIC = 0.0625 µg/mL, 2MIC = 0.125 µg/mL, 4MIC = 0.25 µg/mL), or left untreated as a control. These cells were then cultivated at 37 °C in BHI medium for 24 h. Subsequently, total RNA was extracted from the *S. pneumoniae* cells that had been cultured overnight, both with and without *Coptis rhizome*, using an RNase Mini kit from Qiagen (Toronto, ON, Canada). The concentration of total RNA was determined using an Epoch microplate reader (Winooski, VT, USA).

For cDNA synthesis, a quantitative reverse transcription polymerase chain reaction (qRT-PCR) pre-mix (Pioneer, Korea) was employed. This involved adding random hexamers to the bacterial RNA. The RNA quantification was carried out using a CFX96 Touch™ real-time PCR detection system (Bio-Rad, Singapore), with IQ™ SYBR^®^ Green supermix used for real-time PCR (Bio Rad, Singapore). The gene expression levels of lyt*A* were assessed through qRT-PCR under the following cycling conditions: initial denaturation at 95 °C for 30 s, followed by 45 cycles at 95 °C for 2 s, 56 °C for 5 s, and a final dissociation step at 95 °C for 15 s, followed by 72 °C for 13 s. To compare target gene expression levels, normalization was performed relative to the housekeeping gene STPN in *S. pneumoniae*, using the 2^−ΔΔCT^ method. The primers used for this study can be found in Table 2.

**Table 2:** Primers used for quantitative reverse transcription polymerase chain reaction

Target gene	Primer sequence
lytA-F	5’-ACGCAATCTAGCAGATGAAGCA-3’
lytA-R	5’-TCGTGCGTTTTAATTCCAGCT-3’
STPN-F	5’-CTGCGTTGTATTAGCTAGTTGGTG-3’
STPN-R	5’-TCCGTCCATTGCCGAAGATTC-3’

### Statistical analysis

The analysis of the results was conducted using Graphpad Prism 7, provided by GraphPad Software (La Jolla, California, USA). To compare means across different treatment groups and calculate the *P*-value, one-way analysis of variance (ANOVA) was employed, followed by Tukey’s multiple comparison test. A *P*-value of less than 0.05 was considered statistically significant.

## Results

### Disk diffusion

**Fig. 1:**
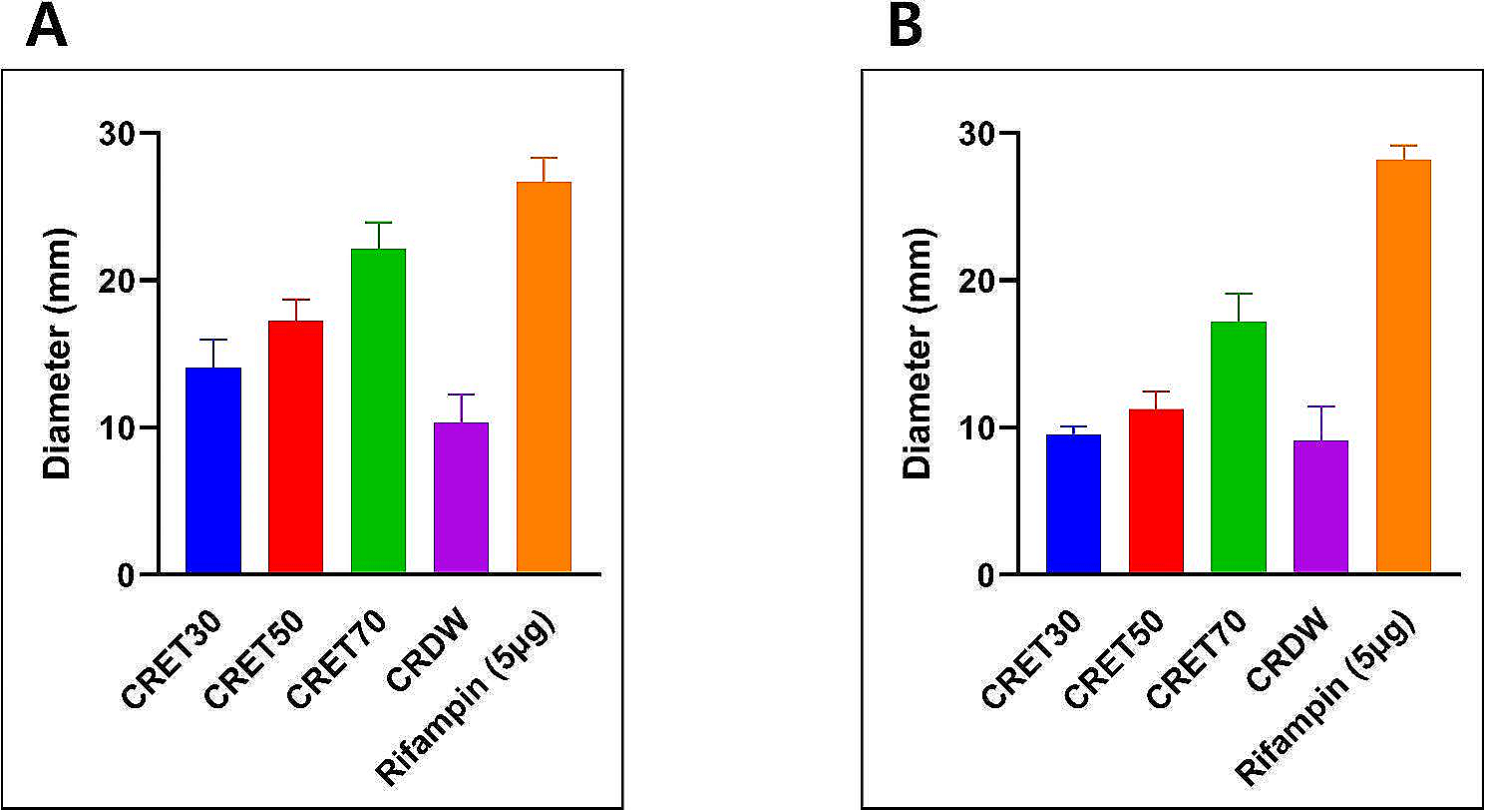
Inhibition effects of *Coptis rhizome *ethanol extract and water extract on two *S. pneumoniae* strains (**A**, ATCC ATCC 49619; **B**, ATCC 70067), showcasing respective inhibition zone diameters. Comparative visualization of antibacterial efficacy through the size of inhibition zones (mm) for each strain and extract. Rifampin (5 µg) was positive control. The data was displayed as the mean ± standard error of the mean (SEM). CRET30, *Coptis rhizome *extracted with ethanol 30%; CRET50, *Coptis rhizome *extracted with ethanol 50%; CRET70, *Coptis rhizome *extracted with ethanol 70%; CRDW, *Coptis rhizome *extracted with distilled water

The Fig. 1 indicated the inhibitory effect of CRET and CRDW on two different strains of *S. pneumoniae*, with respective inhibition zone diameters provided. For the potentially more resistant *S. pneumoniae* strain ATCC 70067, the inhibition zones were comparatively smaller than those for ATCC 49619. Inhibition zones were as follows: CRET30 (14.06 mm for ATCC 49619, 9.57 mm for ATCC 70067); CRET50 (17.26 mm, 11.26 mm); CRET70 (22.17 mm, 17.20 mm); CRDW (10.46 mm, 9.13 mm).

#### MIC

**Fig. 2:**
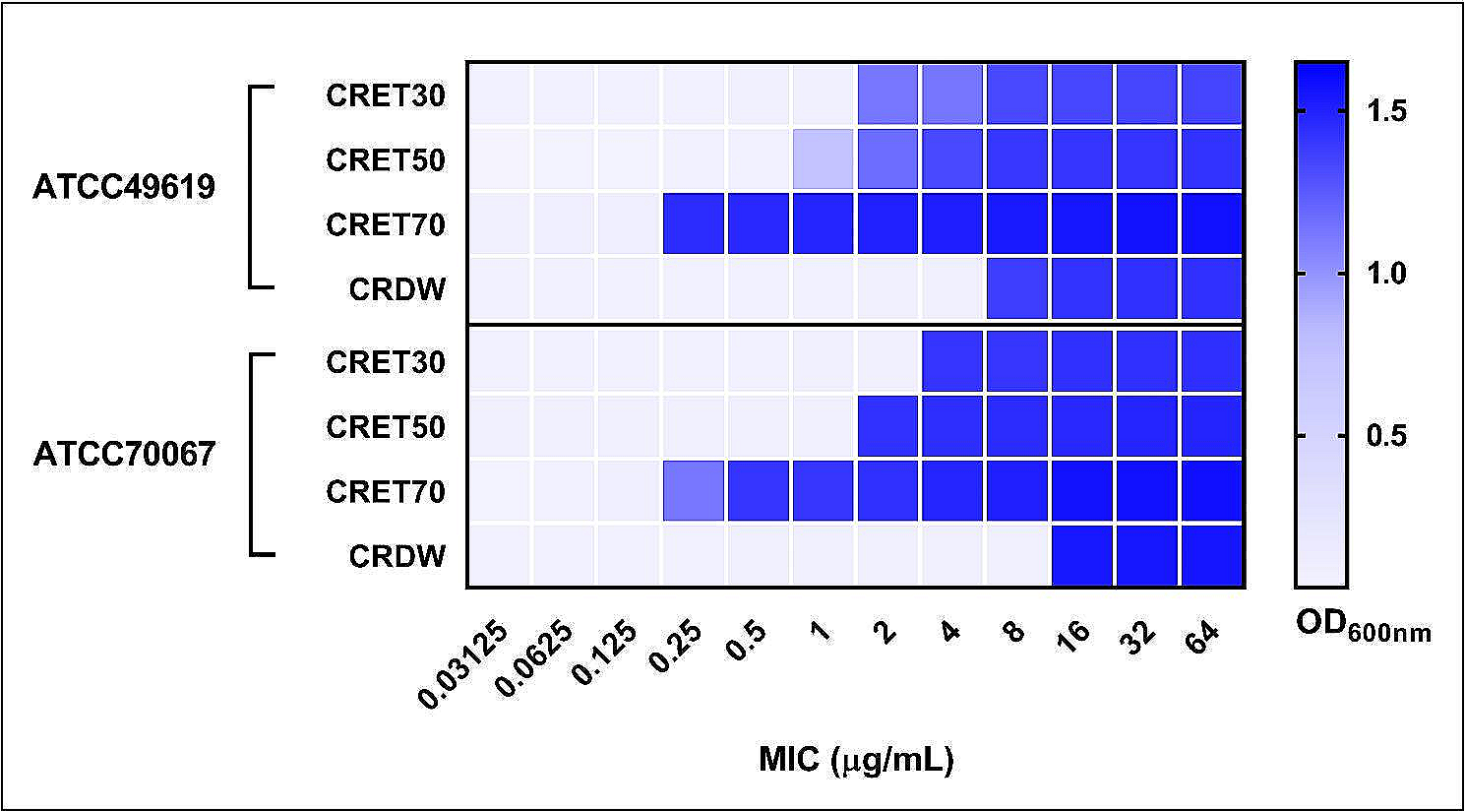
Minimum Inhibitory Concentration (MIC) results for *Coptis rhizome *extracted with either different concentration of ethanol or water against *S. pneumoniae* strains ATCC 49619 (**A**) and ATCC 70067 (**B**). CRET30, *Coptis rhizome *extracted with ethanol 30%; CRET50, *Coptis rhizome *extracted with ethanol 50%; CRET70, *Coptis rhizome *extracted with ethanol 70%; CRDW, *Coptis rhizome *extracted with distilled water

The MIC results of different extracts (CRET and CRDW) against ATCC 49619 (Fig. 2A) and ATCC70067 (Fig. 2B) were described. The MIC results for *S. pneumoniae* ATCC 49619 revealed that the efficacy of the extracts increased with the concentration of ethanol. Specifically, the CRET30 extract had an MIC of 1 µg/mL, which was reduced to 0.5 µg/mL for CRET50, indicating enhanced effectiveness. The CRET70 extract, with the highest concentration of ethanol, showed the greatest potency with an MIC of only 0.125  µg/mL. Conversely, the CRDW exhibited the least antibacterial activity with an MIC of 4 µg/mL, which was the highest among the tested extracts for this bacterial strain. For the presumably more resistant *S. pneumoniae* ATCC 70067, the MIC values were generally higher. The CRET30 extract had an MIC of 2 µg/mL, suggesting it was less effective against this strain compared to ATCC 49619. The MIC for CRET50 was also higher at 1 µg/mL. Notably, the MIC for CRET70 was the same for both strains at 0.125 µg/mL, indicating a strong and consistent antibacterial property. The CRDW’s MIC doubled to 8 µg/mL when tested against ATCC 70067.

### Time kill assay

**Fig. 3:**
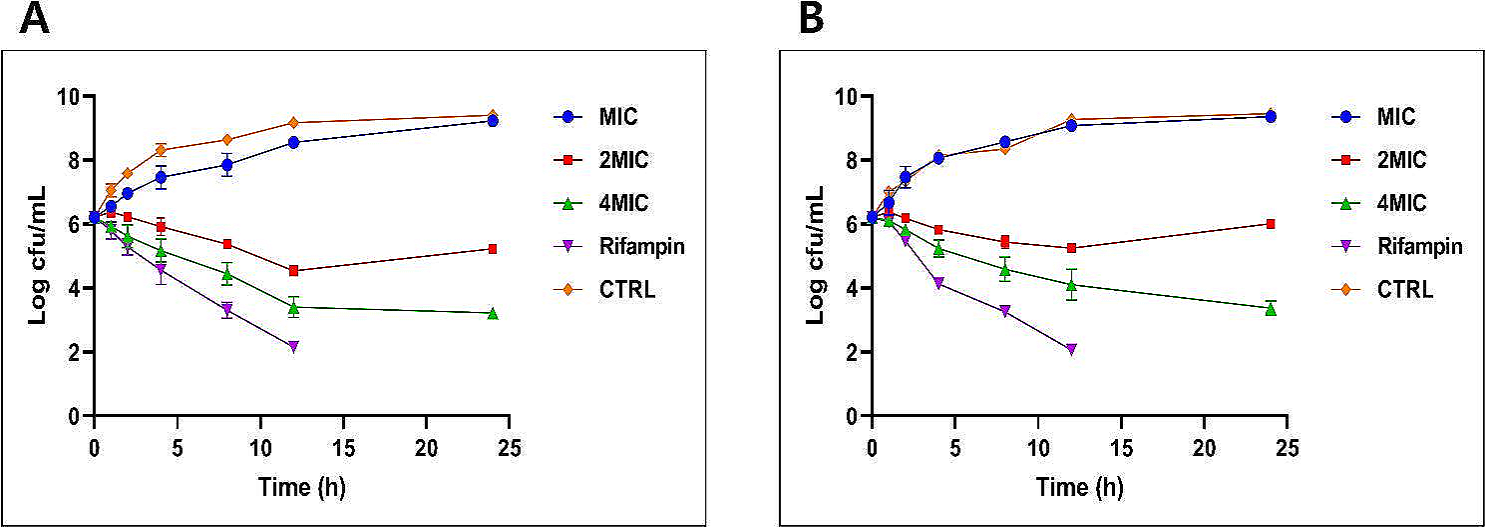
Time-kill curves of *Coptis rhizome *extracted with ethanol 70% at different concentration (1MIC = 0.0625 μg/mL, 2MIC = 0.125 μg/mL, 4MIC = 0.25 μg/mL), or left untreated as a control (CTRL) against *S. pneumoniae* strains ATCC 49619 (**A**) and ATCC 70067 (**B**). Rifampin is regarded as the positive control for comparison. The data was displayed as the mean ± standard error of the mean (SEM)

The time-kill curves of CRET70 against two different strains of *S. pneumoniae*, specifically ATCC 49619 (Fig. 3A) and ATCC 70067 (Fig. 3B), with rifampin being utilized as the positive control for comparison were illustrated. For each strain, CRET70 showed a consistent, concentration-dependent antibacterial effect within a 24 h. With ATCC 49619, administering 4MIC of CRET70 led to a steady reduction in bacterial numbers. A comparable trend was noted for ATCC 70067. In contrast, rifampin demonstrated a more robust antibacterial action, achieving up to a 4-log reduction in bacterial counts. When the effects of 4MIC CRET70 are juxtaposed with those of rifampin, it became apparent that Rifampin exerted a faster and more pronounced antibacterial action within the initial 12 h across both strains. Despite this, CRET70 achieved the substantial bacterial killing over a longer duration.

### Bacterial lysis

**Fig. 4:**
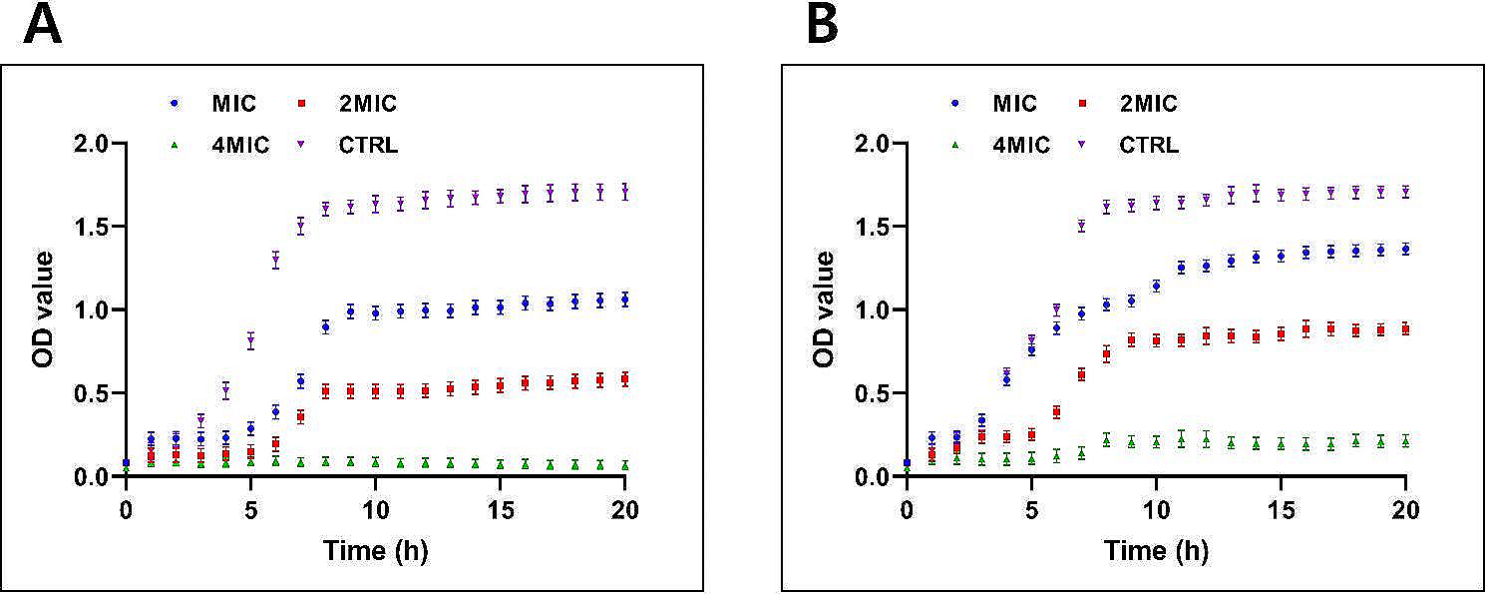
Bacterial lysis following treatment with *Coptis rhizome *extracted with ethanol 70% at different concentration (1MIC = 0.0625 μg/mL, 2MIC = 0.125 μg/mL, 4MIC = 0.25 μg/mL) or left untreated as a control (CTRL) against *S. pneumoniae* strains ATCC 49619 (**A**) and ATCC 70067 (**B**). OD values at 595nm was depicted. The data was displayed as the mean ± standard error of the mean (SEM)

The plotted results in Fig. 4 depicted the optical density (OD) values for *S. pneumoniae* (ATCC 49619 and ATCC 70067) bacterial lysis at a concentration of 4MIC of CRET70 over a time course of 20 h. It was clear that the OD values for the control (CTRL) group increased significantly over time, indicating substantial bacterial growth. In contrast, the OD values for the 4MIC group remained relatively constant and significantly lower than the CTRL throughout the experiment.

### Antibiofilm formation activity

**Fig. 5:**
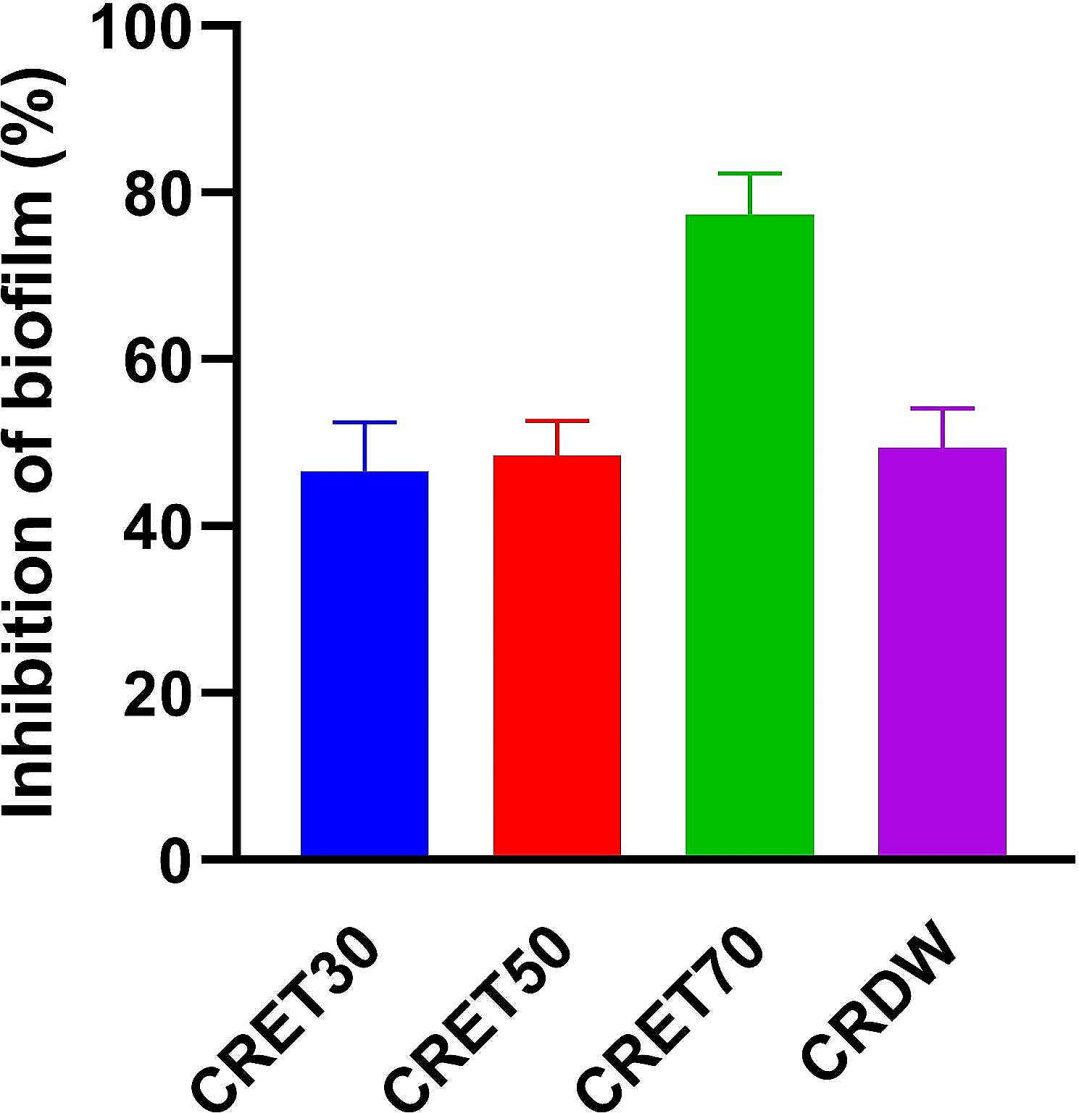
Inhibition of biofilm (%) by the bacteria *S. pneumoniae* ATCC 49619 after treating *Coptis rhizome *extract*. *The data was displayed as the mean ± standard error of the mean (SEM). CRET30, *Coptis rhizome *extracted with ethanol 30%; CRET50, *Coptis rhizome *extracted with ethanol 50%; CRET70, *Coptis rhizome *extracted with ethanol 70%; CRDW, *Coptis rhizome *extracted with distilled water.

The provided data indicated the efficacy of different concentrations of ethanol extracts—CRET30, CRET50, and CRET70—in inhibiting biofilm formation by the bacteria *S. pneumoniae* ATCC 49619, alongside CRDW (Fig. 5). The percentages denoted the average inhibition levels achieved by each treatment. CRET30 demonstrated a biofilm inhibition rate of 46.62%. A slightly higher inhibition was observed with CRET50 which inhibited biofilm formation by 48.44%. In contrast, CRET70 showed a substantially higher biofilm inhibition rate at 77.40%, suggesting that it was considerably more effective than the lower concentrations of ethanol extracts. Meanwhile, the CRDW showed a biofilm inhibition of 49.39%. This rate was comparable to the lower ethanol extracts but is notably less effective than the 70% ethanol extract, indicating that the potency of biofilm inhibition increased with the ethanol concentration in the extracts used.

### lytA gene expression

**Fig. 6:**
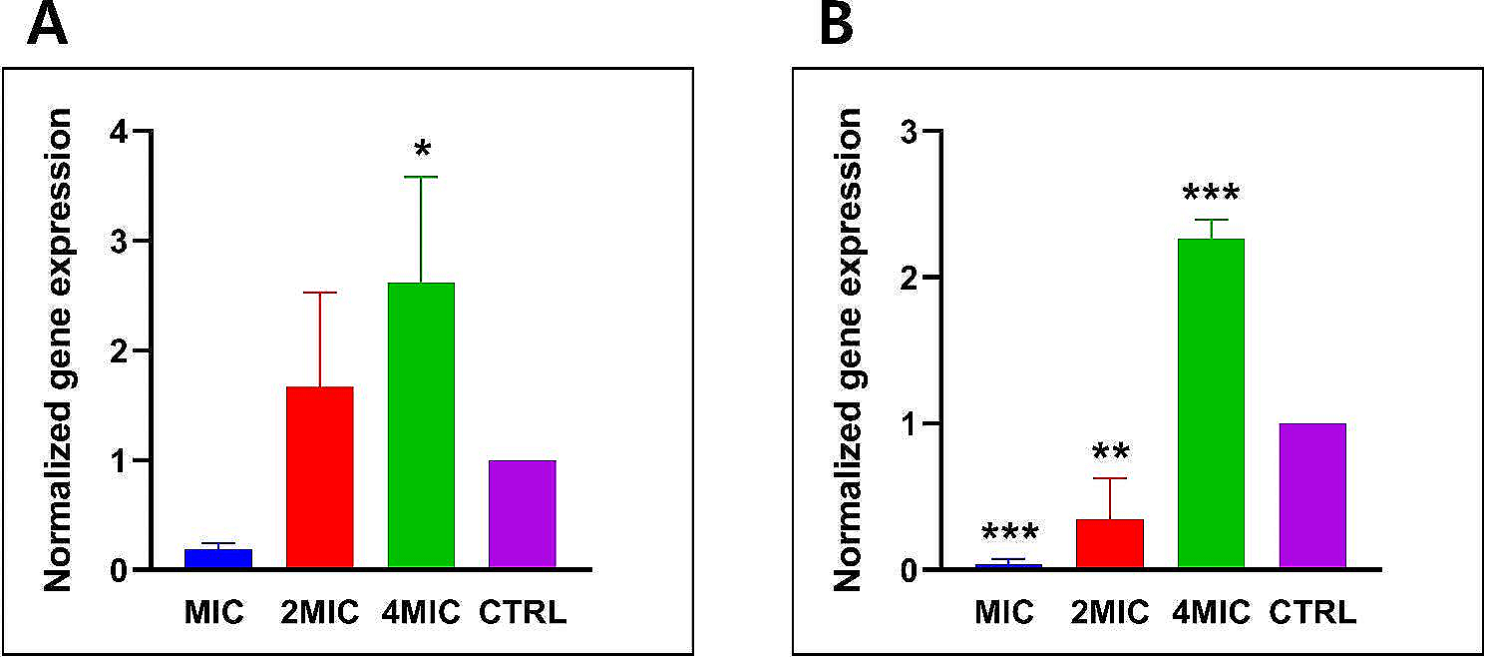
Expression levels of the lytA gene in *S. pneumoniae* strains: standard ATCC 49619 (**A**) and resistant ATCC 70067 (**B**). Analysis of autolysin production under *Coptis rhizome *extracted with ethanol 70% (CRET70) concentrations: 1MIC, 2MIC, 4MIC. The group not treated with the test compound served as the control (CTRL). The values shown were the average differences in expression fold change compared to cells that were not treated. The data was displayed as the mean ± standard error of the mean (SEM) and is based on three separate experiments. *P < 0.05, **P < 0.01,***P < 0.001

Expression levels of the lytA gene, responsible for autolysin production, have been measured in two distinct strains of *S. pneumoniae*, the standard strain ATCC 49619 (Fig. 6A) and the resistant strain ATCC 70067 (Fig. 6B), under varying concentrations of CRET70 at 1MIC, 2MIC, and 4MIC. It was evident across both strains that there was a proportional relationship between the amount of CRET70 administered and the activation of the lytA gene. Specifically, for the standard strain ATCC 49619, expression levels were recorded at 0.19, 1.67, and 2.59 for 1MIC, 2MIC, and 4MIC, respectively. In contrast, for the resistant strain ATCC 70067, the levels were 0.05, 0.35, and 2.27 for the same concentrations. Although a dose-responsive elevation in lytA gene expression was observed in both, the standard strain exhibited a notably higher initial expression at the lowest concentration of CRET70 compared to the resistant variant. The resistant strain demonstrated a need for a higher dose of CRET70, particularly at 4MIC, to reach expression levels seen in the standard strain.

## Discussion

In this study, we have specifically targeted *S. pneumoniae*, a pathogen of considerable clinical concern due to its rapid development of resistance against conventional antibiotics. The focus on this particular bacterium is not merely relevant but essential for advancing the development of targeted therapies, an urgent need given the escalating challenges in treating resistant strains.

While the antibacterial effects of *Coptis rhizome* extracts are well-established, the literature has not thoroughly examined their mechanisms of action against specific strains of *S. pneumoniae*, especially in the context of interactions with key virulence factors such as the lytA autolysin. Our research contributed significantly to this area by exploring how varying concentrations of the extract impact the activity of lytA autolysin, a crucial component in the pathogen’s life cycle and its response to antibacterial agents. By delineating these interactions, our findings provided valuable insights into the potential pathways through which *Coptis rhizome* extracts exert their effects, suggesting a targeted mechanism that could be exploited in therapeutic interventions.

Antibacterial resistance is a significant concern in modern medicine, as it can render standard treatments ineffective, leading to more persistent and potentially dangerous infections. Bacterial autolysis, the self-destruction of bacteria via their own enzymes (autolysins), is intricately linked to this resistance. In addressing antibacterial resistance, the role of medicinal plants has gained prominence. Particularly, the effects of *Coptis rhizome* have become a key area of interest. Consequently, the study has been conducted to assess the antimicrobial effectiveness of *Coptis rhizome* through its ethanol extracts (CRET) and a distilled water extract (CRDW), targeting two specific strains of *S. pneumoniae*: the standard strain ATCC 49,619 and the more antibiotic-resistant variant ATCC 70067. The study also explored the possibility of *Coptis rhizome* interacting with bacterial internal control systems, especially those responsible for autolysis. This research offered valuable insights and sets the stage for future investigations to deepen our understanding of how medicinal plants like *Coptis rhizome* might be used to combat bacterial resistance.

Plant extracts have been utilized in the treatment of *S. pneumoniae*, a bacterium known to cause various infections in humans (Zampini et al. 2012; Yadav et al. 2013). The effectiveness of such treatments is often attributed to the unique compounds found in plants, which can exhibit antibacterial activity, potentially offering an alternative or complementary approach to conventional antibiotics (Bernhoft 2010). This use of plant-based remedies reflects a growing interest in exploring natural sources for managing bacterial infections, especially in the context of rising antibiotic resistance. The antimicrobial activity of *Rubus ulmifolius* have been studied against strains of *S. pneumoniae* that are resistant to commonly used antibiotics (Talekar et al. 2014). This research indicated that *Rubus ulmifolius* can effectively eliminate planktonic pneumococcal cells and interfere with the formation of pneumococcal biofilms. Moreover, the distilled water extract of *Pteruspermum Canescens* has shown effectiveness against multidrug-resistant clinical isolates of *S. pneumonia* (Kadhar Nivas 2020).

*Coptis rhizome*, an herb used traditionally for its medicinal properties, is well-regarded for its range of pharmacological effects due to its active constituents. The major bioactive compounds in *Coptis rhizome* are isoquinoline alkaloids, including berberine, coptisine, palmatine, and jatrorrhizine (Meng et al. 2018). Among these, berberine is the most studied and is known for its potent antibacterial, anti-inflammatory, and antiviral activities. Research suggests that berberine targets bacterial cell walls and interferes with their ability to adhere to host cells, which may partially explain the antibacterial action observed in our study (Zhou et al. 2023a). Furthermore, berberine has been shown to inhibit the formation of biofilms, a key factor in the virulence and antibiotic resistance of many pathogens, including *S. pneumonia* (Zhou et al. 2023b).

In addition to berberine, other alkaloids such as coptisine and palmatine also contribute to the antimicrobial profile of the extract. These compounds have been reported to synergize with berberine, enhancing its efficacy and possibly working through multiple pathways to exert their antibacterial effects. For instance, studies have indicated that coptisine can disrupt bacterial DNA synthesis, while palmatine might interfere with protein synthesis (Tseng et al. 2022). This herbal extract has been applied in diverse medical treatments. Research has shown that using *Coptis rhizome* significantly reduces serotonin levels in the distal colon of rats affected by neonatal maternal separation-induced visceral hyperalgesia, suggesting its use as a potential treatment for irritable bowel syndrome (Tjong et al. 2011). Moreover, *Coptis rhizome* and its main component, coptisine chloride, have demonstrated promising results in combating malaria (Teklemichael et al. 2020). Additionally, Coptidis Rhizoma, along with the alkaloids derived from it, are known for their effectiveness in neuroprotection and as antioxidants, further highlighting the plant’s broad therapeutic potential (Jung et al. 2009). In this study, the results provided valuable information about the antimicrobial effects of CRET and CRDW on two *S. pneumoniae* strains, ATCC 49619 and ATCC 70067, highlighting their possible therapeutic uses. The data indicated that CRET, especially in higher ethanol concentrations, showed promise as an effective treatment option for *S. pneumoniae* infections, including those caused by more resistant variants. Additional exploration into the underlying mechanisms of these substances has also been undertaken, particularly focusing on autolysis.

In our study, we have observed that CRET70 exhibits significantly higher antibacterial activity against *S. pneumoniae* compared to the CRET30 and CRET50 extracts. This enhanced efficacy is likely due to the higher concentration of active phytochemicals, particularly alkaloids, in the CRET70 extract. The extraction process, utilizing varying ethanol concentrations (30%, 50%, and 70% for CRET30, CRET50, and CRET70) plays a critical role in this phenomenon. Ethanol, as a solvent, enhances the solubility and extraction efficiency of alkaloids and other bioactive compounds (Iloki-Assanga et al. 2015; El Mannoubi 2023). Further supporting this, previous study demonstrated that higher concentrations of ethanol improve the extraction of a wider range of alkaloids, which are recognized for their strong antibacterial properties (Kumoro and Hartati 2015). We hypothesize that the elevated ethanol content in CRET70 facilitates a more effective extraction of these potent molecules, thus contributing to the extract’s superior antibacterial performance. This correlation between solvent concentration and phytochemical profile underscores the importance of optimizing extraction conditions to maximize the therapeutic potential of plant extracts.

In the investigation of biofilm inhibition by different concentrations of *Coptis rhizome* extracts, we observed a distinct pattern where the water extract and lower concentrations of ethanolic extracts (30% and 50%) presented similar biofilm inhibitory effects, with a notable increase only at 70% ethanol concentration. This pattern does not align with a typical dose-dependent response, which has been observed in the antibacterial activity of the extracts. This discrepancy raises important considerations about the solubility and bioavailability of bioactive compounds at various ethanol concentrations and the potential influence of ethanol itself. Ethanol, used as a solvent in our extracts, may possess inherent antimicrobial properties that could contribute to biofilm inhibition. The similar efficacy of water and lower ethanol concentrations suggests that the active components within the extracts, possibly alkaloids like berberine, exhibit their biofilm inhibition activity independently of ethanol content. Literature suggests that these compounds can disrupt biofilm formation through mechanisms that might not necessarily correlate with increasing concentrations of ethanol (Almatroodi et al. 2022).

The Autolysin protein, a pivotal enzyme in the life cycle of *S. pneumoniae*, is critical for both the growth and the lysis of the bacterial cell wall (Canvin et al. 1995). Encoded by the *lytA* gene, this protein plays a dual role: it facilitates the separation of daughter cells during division and can also trigger the self-destruction of the cell under certain conditions, an event known as autolysis (Romero et al. 2007). The regulation of Autolysin is thus a key factor in the pathogenicity and survival of *S. pneumoniae*, as its controlled activity is necessary for maintaining cellular integrity and promoting successful colonization or infection.

In this study, we explored the impact of *Coptis rhizome* extract on the activity of lytA autolysin in *S. pneumoniae*. Our findings revealed varied expression levels of lytA across different MIC levels, posing intriguing questions about the underlying mechanisms. Recognizing that lytA is constitutively expressed and generally sufficient for pneumococcal cell lysis without the need for overexpression, our results suggested that the *Coptis rhizome* extract may modulate this autolysin’s activity through mechanisms other than direct induction.

The literature suggested that traditional beta-lactam antibiotics do not require increased lytA production but rather facilitate its activity through interference with cell wall synthesis, leading to autolysis (Mellroth et al. 2012). Contrary to this, the *Coptis rhizome* extract appeared to interact differently, potentially influencing the regulatory or signaling pathways that control lytA activity. The reduction in lytA expression observed at lower concentrations (1MIC and 2MIC) could indicate a stress response triggered by the extract, which transiently suppresses the expression or alters the activation of lytA (Vieira et al. 2024). Conversely, the increase in lytA expression at a higher concentration (4MIC) suggests a threshold effect where higher doses of the extract might engage different cellular pathways or regulatory mechanisms. Therefore, in this context, the influence of *Coptis rhizome* on the expression of the lytA gene is particularly noteworthy. *Coptis rhizome* was observed to upregulate the expression of the lytA gene. This suggested that compounds within *Coptis rhizome* may interact with the bacterial regulatory mechanisms that control autolysis. The overexpression of lytA could potentially lead to an increase in autolysin production, which in turn may enhance the rate of bacterial lysis (Chapot-Chartier 2010). Such an increase could disrupt the balance between cell wall synthesis and degradation, leading to a reduction in viable bacterial cells and thereby mitigating infection. This could be particularly useful against antibiotic-resistant strains, offering an alternative mechanism of action that disrupts bacterial cell wall integrity. Further research into the active components of *Coptis rhizome* and their molecular interactions with *S. pneumoniae* could pave the way for new antibacterial strategies and enhance our understanding of bacterial autolysis as a target for therapeutic intervention.

In conclusion, this study highlighted the potential of medicinal plants, specifically *Coptis rhizome*, in combating antibacterial resistance. By employing various ethanol concentrations to extract *Coptis rhizome*, it was determined that CRET70 exhibited potent antibacterial activity, demonstrated by a 2-log reduction against both standard and resistant strains of *S. pneumoniae*. Notably, the study found an overexpression of the autolysin lytA gene in response to CRET70 treatment. This suggested a significant impact of *Coptis rhizome* extracts on bacterial cell mechanisms, offering a promising avenue for addressing antibiotic resistance.

## Data Availability

The data generated or analyzed during this study are included in this article.
